# Creation and observation of Hopfions in magnetic multilayer systems

**DOI:** 10.1038/s41467-021-21846-5

**Published:** 2021-03-10

**Authors:** Noah Kent, Neal Reynolds, David Raftrey, Ian T. G. Campbell, Selven Virasawmy, Scott Dhuey, Rajesh V. Chopdekar, Aurelio Hierro-Rodriguez, Andrea Sorrentino, Eva Pereiro, Salvador Ferrer, Frances Hellman, Paul Sutcliffe, Peter Fischer

**Affiliations:** 1 Materials Sciences Division, Lawrence Berkeley National Laboratory, Berkeley, CA USA; 2 Physics Department, UC Santa Cruz, Santa Cruz, CA USA; 3 Department of Physics, University of California, Berkeley, Berkeley, CA USA; 4 The Molecular Foundry, Lawrence Berkeley National Laboratory, Berkeley, CA USA; 5 Advanced Light Source, Lawrence Berkeley National Laboratory, Berkeley, CA USA; 6 Department of Physics, University of Oviedo, Oviedo, Spain; 7 ALBA Synchrotron, Cerdanyola del Vallès, Spain; 8 Department of Mathematical Sciences, Durham University, Durham, UK

**Keywords:** Magnetic properties and materials, Magnetic properties and materials

## Abstract

Among topological solitons, magnetic skyrmions are two-dimensional particle-like objects with a continuous winding of the magnetization, and magnetic Hopfions are three-dimensional objects that can be formed from a closed loop of twisted skyrmion strings. Theoretical models suggest that magnetic Hopfions can be stabilized in frustrated or chiral magnetic systems, and target skymions can be transformed into Hopfions by adapting their perpendicular magnetic anisotropy, but their experimental verification has been elusive so far. Here, we present an experimental study of magnetic Hopfions that are created in Ir/Co/Pt multilayers shaped into nanoscale disks, known to host target skyrmions. To characterize three-dimensional spin textures that distinguish Hopfions from target skyrmions magnetic images are recorded with surface-sensitive X-ray photoemission electron microscopy and bulk-sensitive soft X-ray transmission microscopy using element-specific X-ray magnetic circular dichroism effects as magnetic contrast. These results could stimulate further investigations of Hopfions and their potential application in three-dimensional spintronics devices.

## Abstract (web summary)

In three dimensions, magnetic Skyrmions can form extended strings. By connecting the ends of a Skyrmionic string, a magnetic Hopfion is formed. Here, the authors present experimental evidence for the appearance of Hopfions, a topological soliton defined by a Hopf number, in a magnetic system.

## Introduction

In classical field theories, topological solitons describe stable, particle-like objects that have a finite mass and a smooth structure^1^. Whereas first approaches focused on problems in high-energy physics, recently this concept has seen significant attention in condensed matter research, specifically in ferromagnetic^2,3^ or ferroelectric materials^4^. There, the smoothly varying vector field is the magnetization or the polarization of the material, respectively. The topological nature of the field is described by a topological charge that describes the continuous winding of the vector field, i.e., the magnetization in a magnetic topological soliton.

Prominent examples are magnetic skyrmions, which are two-dimensional topologically protected spin textures, where the topological charge, the skyrmion number, is defined as $N_{sk} = \frac{1}{{4\pi }}{\int\!\!\!\!\!\int} {\left. {d^2r{\mathbf{m}} \cdot \left( {\frac{{\partial {\mathbf{m}}}}{{\partial {\mathrm{x}}}} \times \frac{{\partial {\mathbf{m}}}}{{\partial y}}} \right)} \right)}$ with **m** being the unit vector of the magnetization. The spin textures in ferromagnets are the result of competing interactions, such as exchange and magnetic anisotropy, where the former prefers a parallel alignment of neighboring spins, and the latter favors certain orientation, e.g., given by the crystallography of the materials. Mathematically, in most condensed matter systems, localized structures, such as magnetic skyrmions, would rapidly collapse into linear singularities (Hobart–Derrick theorem)^5^. However, in magnetic materials with broken inversion symmetry, which can be achieved, e.g., through a chirality in the crystallographic structure or at interfaces in low-dimensionality thin films, an additional asymmetric exchange interaction, the so-called Dzyaloshinksi–Moriya interaction (DMI), enables the stabilization of localized magnetic states such as skyrmions with finite sizes. The spin texture in magnetic skyrmions can be described by a continuous rotation of the magnetization in the radial direction from the core at the center pointing antiparallel to the orientation far away from the center. The stability of magnetic skyrmions and their high mobility enabling them to be driven through a material with relatively low current densities make them promising candidates for future high-density, high-speed, and low-power spintronics applications. Although for most of the current research on magnetic skyrmions, they are considered to be two-dimensional topological solitons; in real systems, they are nanoscale cylinders, and their two-dimensional treatment assumes a rigid three-dimensional structure. Other three-dimensional spin textures, such as chiral bobbers^6,7^ or skyrmion strings^8^, which have recently been found in spin systems, have a robust and flexible three-dimensional structure exhibiting, e.g., nontrivial dynamic responses^9^.

**Fig. 1:**
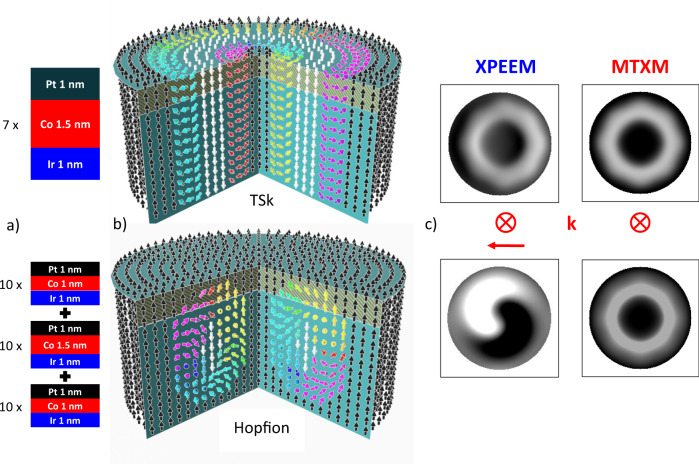
Spin texture of a 2π TSk and a *Q* = 1 Hopfion. **a** Schematics of the Ir/Co/Pt multilayer systems (S7, S30) used in this study. **b** Schematical drawing of the spin texture of a 2π TSk (top) and a symmetric *Q* = 1 Hopfion (bottom). The yellow-shaded region near the top indicates the approximate depth sensitivity of X-PEEM. **c** Simulated X-PEEM (left) and MTXM (right) signals for a TSk (top) and a Hopfion (bottom). The direction of the photon angular momentum (k) is indicated for X-PEEM (in-plane and out-of-plane) and MTXM (only out-of-plane).

A generalization of magnetic skyrmions into the third dimension leads to more complex and diverse topological solitons, including rings, knots, and links. Hopf solitons or Hopfions (Fig. 1b, bottom) are such three-dimensional topological solutions, particularly knot solitons, that can be classified by another topological invariant, the Hopf number (*Q*_*H*_), which in real space can be expressed as $Q_H = - {\int} {{\mathbf{B}} \cdot {\mathbf{A}}d^3r}$ with ***B*** being the emergent magnetic field from the spin texture and **A** the magnetic vector potential^10^. The Hopf number distinguishes the topology in different Hopfions and can be geometrically interpreted as the linking number of any two closed-loop regions in 3D real space that contains spins pointing in the same direction^11,12^. Consequently, a Hopfion can be viewed as a closed loop of a twisted skyrmion string^13^. Whereas stable Hopfions with sizes in the micrometer regime have been experimentally observed, e.g., chiral ferromagnets and liquid crystals^14^, the realization of stable Hopfions in chiral magnets or magnetic nanostructures, has been elusive so far, but given the enormous progress in understanding the static and dynamics of magnetic skyrmions in low-dimensional magnetic systems and their exploration toward novel spintronics applications, an expansion into the third dimension with magnetic Hopfions is very attractive and may allow the discovery of novel physical phenomena. Since Hopfions are truly three-dimensional in nature, and therefore there is no two-dimensional approximation, advances in nanoscale three-dimensional synthesis and specifically characterization are essential to make progress.

Theoretical models and simulations predict that Hopfions can be stabilized in a variety of magnetic systems, including frustrated magnets^15^, and, most promisingly, chiral magnets hosting target skyrmions (TSks)^12^. Target skyrmions^16–18^ are extended skyrmionic spin textures, where the magnetization rotates multiple times (*n*π)^19^ and which have a topological charge that toggles from 0 to 1 for even and odd number of rotations *n*, respectively. They have been found in nanostructures of magnetic multilayers with strong DMI and perpendicular magnetic anisotropy (PMA), and are expected to host stable Hopfions by tuning the PMA at their interfaces. In particular, a 2π TSk transforms into a Hopfion when the magnetization at the top and bottom of the TSk changes to align with the magnetization at the center of the TSk. Figure 1b displays 3D schematics of the spin textures indicating characteristic differences between the TSk and the Hopfion near the top and bottom of the structures.

In this work, we report an experimental study of bespoke magnetic multilayers following theoretical predictions so as to create magnetic Hopfions. To characterize their three-dimensional spin textures that in particular distinguish Hopfions from target skyrmions, we record magnetic images with ~20–30-nm spatial resolution with surface-sensitive X-ray photoemission electron microscopy and bulk-sensitive soft X-ray transmission microscopy using element-specific X-ray magnetic circular dichroism effects as magnetic contrast. A comparison with simulated data provides strong evidence that magnetic Hopfions have been successfully created in those systems.

## Results and discussion

### Synthesis of Hopfions in multilayer systems

We fabricated via magnetron sputtering two magnetic multilayer systems that were designed to host either a TSk or a magnetic Hopfion and will be in the following referred to as S7 (TSk) and S30 (Hopfion), respectively (Fig. 1a). The multilayer structure of S7 consists of {Ir(1 nm)/Co(1.5 nm)/Pt(1 nm)}x7, i.e., 7 layers of a PMA stack, and similarly, S30 consists of {Ir(1 nm)/Co(1 nm)/Pt(1 nm])}x10 + {Ir(1 nm)/Co(1.5 nm)/Pt(1 nm)}x10 + {Ir(1 nm)/Co(1 nm)/Pt(1 nm)}x 10, i.e., 30 layers of a PMA stack with a varying Co thickness (Fig. 1a). As a result of the interface- induced PMA by Ir and Pt into Co, which is larger for the thinner Co layer, the PMA value in S7 with the 1.5-nm-thick Co layer is reduced to ~25–30% compared to S30 with a 1-nm-thick Co layer. To confine locally the spin textures and to harness also shape anisotropy, which supports the formation of TSks and Hopfions, nanodisks with a thickness of 24.5 nm (S7) and 95 nm (S30), respectively, and with varying diameters ranging from 2000 nm down to 100 nm, were fabricated using electron-beam lithography (EBL). The domain structure of S7 in a disk with a diameter of 1500 nm as seen with X-PEEM is shown in Supplementary Fig. 1, and is found to be similar to the TSk textures observed previously with MTXM^17^.

### Imaging spin textures with X-ray spectromicroscopy

To identify and distinguish the characteristic three-dimensional nature of the spin textures specifically between TSks and Hopfions (Fig. 1b), we took an experimental approach to combine two advanced magnetic X-ray microscopy techniques each providing spatial resolutions that are typically in the 20–30-nm range, and both using X-ray magnetic circular dichroism (XMCD) as the element-specific magnetic contrast mechanism. X-ray photoemission electron microscopy (X-PEEM) provides information about the spin texture close to the surface due to the limited escape depth of the detected secondary photoelectrons, while magnetic soft X-ray transmission microscopy (MTXM) images the local spin texture averaged throughout the thickness of the system. Further, XMCD measures the projection of the magnetization onto the photon propagation directions, which not only allows distinguishing in-plane vs out-of-plane components of the local magnetization, but in principle enables also a reconstruction of the 3D spin texture by recording images at various incidence angles of the X-rays. Figure 1c shows simulations of experimental X-PEEM and MTXM images for a TSk and a Hopfion at a specific incidence angle, respectively. The MTXM images for the TSk and Hopfion exhibit both a pronounced black area at the center (“bulls-eye”), surrounded by a uniform bright ring and again a smaller black ring at the perimeter indicating that for a *Q* = 1 Hopfion and 2π TSk MTXM images cannot distinguish between those two spin textures. However, the simulated X-PEEM images (Fig. 1c, left column) show already at a single incidence angle-distinct differences between the two spin textures. The TSk (Fig. 1c, top) exhibits a similar ring pattern to the MTXM image, whereas the Hopfion PEEM image exhibit a “ying-yang”-like pattern of a black-and-white structure each filling about half of the disk.

**Fig. 2:**
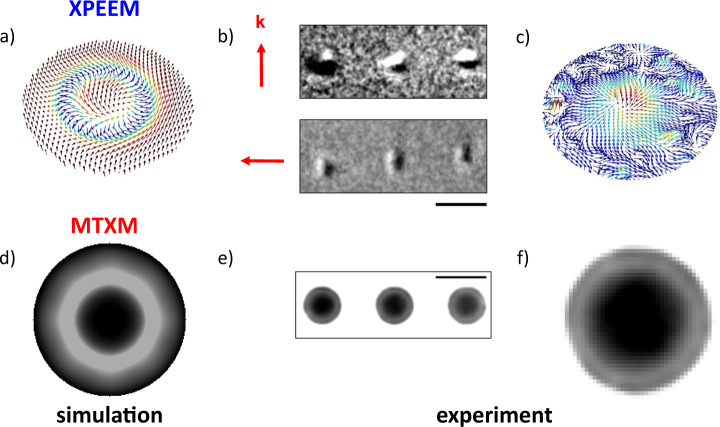
Simulation and experimental results for X-PEEM and MTXM. **a** Simulation of the full 3D spin textures expected in X-PEEM. **b** Rows of three S30 nanodisks with 400-nm diameter observed with X-PEEM under *θ* = 0° and *θ* = 90°. **c** 3D spin texture reconstructed from experimental X-PEEM images. **d** Simulation of the MTXM image. **e** MTXM images of a row of three S30 nanodisks as in (**b**) showing a reproducible characteristic spin texture. **f** MTXM image of the identical disk as in (**c**). Color coding in (**a**) and (**c**): red points up, light-blue points down. Scale bars in (**b**) and (**e**) are 500 nm.

Our experimental results obtained with the 400-nm-diameter pillars of the S30 sample are shown in Fig. 2. The X-PEEM data were taken at PEEM3 (BL 11.0.1.1)^20^ at the Advanced Light Source in Berkeley, CA, and the MTXM images shown were recorded at the MISTRAL beamline (BL-09)^21^ at the ALBA synchrotron in Barcelona/Spain. Both X-PEEM and MTXM data were recorded at remanence after the samples had been saturated by an external magnetic field. This approach is consistent with previous studies on TSks^17^. The MTXM images were recorded with the X-ray propagation direction orthogonal to the sample surface to maximize the XMCD contrast from out-of-plane magnetization. The X-PEEM images were recorded at an incidence angle of 30° with respect to the sample surface. To obtain a full 3D reconstruction of the spin textures in the surface region, X-PEEM images were recorded with the sample rotated around an axis normal to the surface by 0, 90, and 180°. Due to technical limitations, the MTXM images could not be recorded at varying incidence angles of the X-ray beam relative to the sample’s surface; therefore, those data cannot support any 3D characterization of the spin textures, but they allow the exclusion of other spin textures, e.g., torons (see Supplementary Fig. S2).

Figure 2a shows the simulation of the full 3D spin texture as it is expected in the X-PEEM image. The experimental X-PEEM data shown in Fig. 2b exhibit a characteristic black–white feature as expected from the simulation at a single incidence angle (Fig. 1c). The corresponding X-PEEM at a rotation of 90° confirms the expected rotation of this black–white pattern. Combining the X-PEEM images from all three incidence angles retrieves the reconstructed 3D rendering of S30 shown in Fig. 2c. Comparing the experimental X-PEEM data (Fig. 2c) with the simulated X-PEEM data (Fig. 2a), there is a wide range of agreement between the experiment and the simulated data. The experimental data clearly show a red inner core surrounded by a blue ring in agreement with the simulations. The agreement with simulations for the features closer to the rim of the structures is less obvious. The data seem to have a higher noise level than in the center, but this is most likely due to both the limited spatial resolution and the difficulties with X-PEEM to image edge structures that rise sharply above the surface leading to a “focusing” effect^22^. The distinct characteristics of the X-PEEM 3D spin texture for a magnetic Hopfion are a strong indication that the S30 system hosts magnetic Hopfion textures.

The experimental MTXM images recorded at the exact same disk as the X-PEEM images are shown in the bottom row of Fig. 2. The characteristic bulls-eye structure (Fig. 2c), which consists of an out-of-plane magnetization locally confined in the center of the disk and surrounded by a smooth magnetization pointing into the opposite directions, is clearly visible. Since MTXM does not face the same challenge with imaging edge structure as X-PEEM, the faint black ring at the perimeter that can be seen in Fig. 2f seems to be even resolved, although this could also be a result of interference fringes that tend to occur in MTXM at the boundaries of well-defined structures. In conclusion, although the MTXM alone cannot distinguish between a TSk and a Hopfion, the experimental observation is compatible with the simulation, and the full 3D reconstruction of the X-PEEM data, despite its limitation toward the rim of the structures, exhibits characteristic spin textures that are in agreement with the simulation and therefore can be seen as it is a strong indication to the existence of a magnetic Hopfion. Although it is not solid experimental proof, this conclusion supports the theoretical predictions in those magnetic multilayers.

### Theoretical simulations of Hopfions

**Fig. 3:**
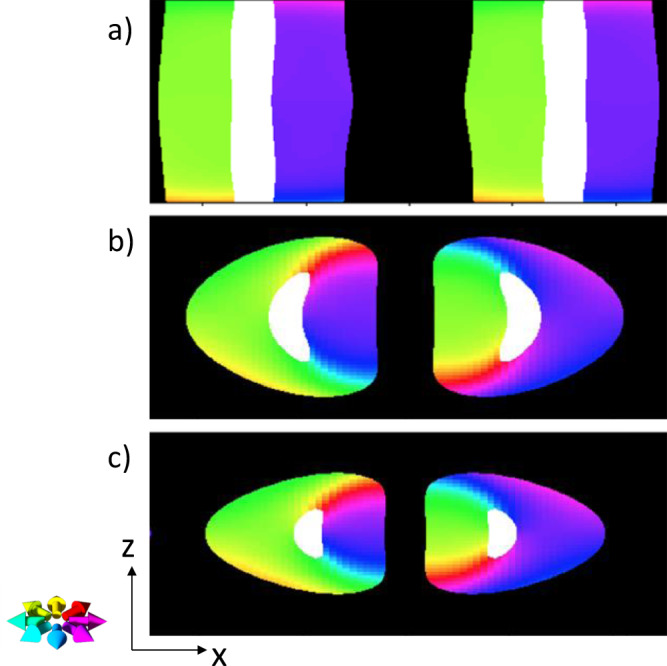
Theoretical simulations. A slice along the *x*–*z* plane (*z* along the disk height) of a variable PMA magnet with DMI. Color code as in Fig. 1b, i.e., black corresponds to up, and white to down, and the color wheel refers to the in-plane (*x*–*y* plane) magnetization direction. The PMA in the central third of each simulated layer is lower than the PMA of the top, bottom third of each simulation. The ratio of high-to-low PMA is (**a**) 2:1, (**b**) 3:1, and (**c**) 4:1.

To study in detail the variation of the spin textures as a function of PMA, further theoretical simulations are presented in Fig. 3, which show that sandwiching a TSk hosting layer between two layers with high PMA will stabilize a Hopfion for all PMA values as long as the out-of-plane components of the TSk do not reach the surface of the magnetic material. With decreasing PMA, the Hopfion will extend further into the top and bottom PMA layers until finally target skyrmion forms. These theoretical predictions are consistent with our experimental X-PEEM observations in S30 showing that the PMA domain structure does not extend throughout the magnetic multilayer system.

In conclusion, we have presented an experimental and theoretical study of the stabilization of magnetic Hopfions that were created in magnetic Ir/Co/Pt multilayers with tailoring their PMA values, and shaped into nanoscale disks. Combining the experimental observations of the existing spin textures from surface-sensitive X-ray photoemission electron microscopy and bulk-sensitive soft X-ray transmission microscopy, we have observed in 400-nm-diameter disks characteristic features that are within the experimental limitations of the imaging techniques consistent with Hopfion spin textures as expected from theory. The out-of-plane bulls-eye texture located at the center of the nanodisk does not extend through the whole thickness of the sample, and a 3D rendering of the magnetic contrast at the surface indicates that a *Q* = 1 Hopfion was stabilized by interfacial DMI.

Although our combined X-PEEM and MTXM results provide strong indications that magnetic Hopfions have been stabilized in the magnetic multilayers as predicted from theory, our data do not constitute a direct and full 3D reconstruction of the spin texture with nanometer spatial resolution, which would be needed to claim that without ambiguity. Despite various encouraging developments, such techniques are not yet available, but there is the potential that with further advances of X-ray microscopies harnessing the coherence at next-generation coherent X-ray sources, this could come within reach.

Nevertheless, we expect that our results will not only stimulate further experimental and theoretical studies to search for Hopfions in magnetic nanostructures with different topologies and Hopf numbers, but should also open the door to exploiting novel phenomena in magnetic Hopfions, including Hopfion dynamics^23,24^ that could lead to new applications in three-dimensional spintronic applications^25,26^. For example, compared to magnetic skyrmions, the vanishing gyrovector in Hopfions would enable racetrack devices, which are not impacted by undesirable Hall effects^23^.

## Methods

### Sample fabrication

The design of the samples took into consideration various specific requirements of the X-ray spectromicroscopy techniques that were primarily used for their characterization, i.e., magnetic transmission soft X-ray microscopy (MTXM) and X-ray photoemission electron microscopy (X-PEEM). To provide sufficient transmission of the soft X-rays between 700- and 800-eV photon energy, commercially available 100-nm thin amorphous silicon nitride (a-SiN_x_) membranes were chosen as substrates. They exhibit an 85% transmission at the Co L_3_ edge at 778 eV, where most of the measurements were taken. To avoid potential electrostatic interference with the edges of the sample holder in the X-PEEM instrument, large 1 × 1-cm Si frames were chosen with a 3 × 3-mm Si_3_N_4_ membrane window in the center.

The magnetic multilayers were patterned into nanopillars of varying diameters ranging from 2000 to 100 nm using EBL at the Molecular Foundry in Berkeley, CA. Polymethyl methacrylate (PMMA) resist with aquaSAVE™, a conductive polymer used to alleviate charging problems, was exposed with a Vistec VB300 EBL tool and then developed in a 3:1 ratio of isopropyl alcohol: methyl isobutyl ketone (IPA:MIBK).

After development, magnetic multilayers were deposited via DC magnetron sputtering using a five-gun AJA ATC2000 confocal magnetron sputtering system with a liquid N_2_ cryojacket achieving a base pressure <8 × 10^−8^ torr. Layers were deposited with an Argon pressure of 1.3 mtorr and at a rate of 0.04 nm/s. The roughness of the multilayers was measured with atomic force microscopy (AFM) showing a smooth surface with an RMS < 0.1 nm.

After the deposition of the magnetic multilayers on the developed PMMA, an extended liftoff in dichloromethane was done, with the membranes upside-down to alleviate strain. A 2-nm Pt overlayer was deposited after liftoff to alleviate charging problems due to the low electrical conductivity of a-SiN_x_ with X-PEEM.

To characterize the macroscopic magnetic properties of the magnetic multilayers, M(H) hysteresis curves were measured using a Lakeshore model 7400 vibrating sample magnetometer (VSM). They matched well with Ir/Co/Pt multilayers that host TSk^17^, and with published work by others^27^. To confirm the increase in PMA for the Co 1-nm multilayers, a reference system {Ir(1 nm)/Co(1.5 nm)/Pt(1 nm)}x10, {Ir(1 nm)/Co(1 nm)/Pt(1 nm)}x10 multilayers was used (not shown in this paper). A first-order approximation of PMA, i.e., for $K_u$ using $H_k = 2K_u/M_s$, yields a value for $K_u$ in the 1-nm Co stack that is 3–4 times larger than in the 1.5-nm Co stack that is in agreement with simulations where Hopfions are stabilized (Fig. 3).

### Magnetic soft X-ray microscopy

To characterize the distinct three-dimensional spin texture in TSks and Hopfions, two complementary advanced soft X-ray microscopy techniques were used, X-ray photoelectron emission microscopy (X-PEEM) and magnetic transmission X-ray microscopy (MTXM). Both use X-ray magnetic circular dichroism (XMCD) as an element-specific magnetic contrast mechanism. Large XMCD effects occur in the vicinity of element-specific inner core X-ray absorption edges, e.g., at the L-edges of a transition metal such as Fe, Co, Ni, or the M edges of rare earth systems. XMCD measures the dependence of the X-ray absorption coefficient of circularly polarized X-rays relative to the direction of the magnetization of a ferromagnetic material and scales with the projection of the magnetization onto the photon propagation direction of the X-rays. In combination with a laterally resolved detection of this XMCD contrast, images of the magnetic domain structures can be recorded in magnetic X-ray microscopies.

In X-PEEM, the incoming monochromatic X-ray beam illuminates the specimen at a shallow angle, which for the PEEM3 system at the Advanced Light Source in Berkeley CA, where the data for this research were taken, amounts to 30° to the surface of the sample. Therefore, X-PEEM has an increased sensitivity to the in-plane component, although also the out-of-plane component can be detected. A rotating sample holder allows for in situ rotation of the sample to determine the full 3D spin texture near the surface. X-PEEM detects the secondary photoelectrons generated in the X-ray absorption process, which leave the surface and are then transmitted through an electronic optical system onto at 2D CCD devices, which records the emitted electrons. Since the escape depth of those secondary electrons is limited to about 5 nm, X-PEEM images the spin texture from that depth only. With advanced aberration-corrected PEEM systems, a spatial resolution down to ~20 nm has been reported^28^. Magnetic X-PEEM experiments are generally performed at remanence to avoid serious distortion of the electron beams by varying external magnetic fields.

The MTXM system is built in analogy to optical microscopy consisting of the source, a condenser optics, a high-resolution objective lens, which for X-rays is a Fresnel zone plate (FZP), and a two-dimensional back-illuminated CCD detector. At the MISTRAL beamline^21^, where the MTXM images were recorded, the optical system consists of a PGM monochromator that provides monochromatic X-rays. A capillary condenser system collimates the photon and provides uniform illumination of the specimen. An image of the transmitted photons is formed by the downstream high-resolution microzone plate (MZP) of 25-nm outermost zone width and directly recorded by a CCD device. The spatial resolution in an MTXM is primarily determined by the quality of the MZP and has been shown to be able to less than 10nm^29^. The MZP at MISTRAL provides ~20–30-nm spatial resolution. MTXM detects directly the transmitted photons and therefore, if the incident photon intensity is known, the actual X-ray absorption coefficient can be derived following Beer’s Law. In normal incidence, the XMCD contrast in MTXM detects the out-of-plane magnetic component only. In order to image in-plane components, the sample is typically rotated around an axis perpendicular to the X-ray propagation direction. In general, MTXM can image magnetic domains in varying external magnetic fields; however, for this study, all images were recorded in remanence.

To allow for a direct comparison of the X-PEEM and MTXM images, the same sample, which was fabricated on X-ray-transparent a-SiN_x_ membranes, was used for both the X-PEEM and the MTXM experiments, to ensure that the identical structure was imaged with both techniques. Both X-PEEM and MTXM are full-field X-ray microscopy techniques covering a field-of-view of several µm in a single image. Typical image acquisition times were a few seconds per image for both MTXM and X-PEEM. The spectral resolution for both X-PEEM and MTXM was sufficiently high at about ΔE/E ~10^3^. All measurements were performed at room temperature.

X-PEEM and MTXM images were recorded as 2 dim arrays of 1024 × 1024-px CCD data.

### Data analysis

The experiments shown in this paper were recorded at the Co L_3_ absorption edge. The MTXM data were taken at fixed circular polarization, and standard image processing techniques were applied to take into account the much larger count rate in the areas surrounding the disks due to the much lower X-ray absorption than in the disk itself.

To separate the in-plane from the out-of-plane components in the X-PEEM images, four series of images were recorded at each location and for each incident angle θ: left circularly polarized X-rays at the resonant Co L_3_ edge (L_ON), 2) left circularly polarized light below the Co resonance at 770 EV (L_OFF) and vice versa for right circularly polarized X-rays (R_ON and R_OFF, respectively).

Normalized XMCD images were derived according to Image(*θ*) = (L_ON)/(L_OFF) − (R_ON/R_OFF), which allows for quantitative comparison of the magnetization when imaged at different angles. Since this normalization process removes the nonmagnetic background in X-PEEM, the surrounding areas of the disks appear gray, whereas they appear white (=saturated) in the MTXM images.

The variation of incident angles in X-PEEM, i.e., images measured at 0, 90, and 180°, where 0° is chosen as an arbitrary value, allows to extract the out-of-plane component: Image(OOP) = Image(0°) + Image(180°), since the in-plane component points in the opposite direction and therefore cancels out. Similarly, the combinations Image(0°) – Image(OOP)/2 and Image (90°) − Image(OOP)/2 provide the in-plane components. The combination of in-plane and out-of-plane components was used to finally retrieve the full 3D orientation of the spin textures in the X-PEEM images.

Imaging isolated nanostructures in MTXM and X-PEEM can create artifacts at the boundaries originating from different sources. In X-PEEM, charge can build up near the edge of isolated structures, resulting in a reduced area of magnetic contrast compared to the physical nanostructure. In MTXM, unless the XMCD can be precisely and without image distortion modulated by polarization reversal, the abrupt change in transmission at the edge of the nanostructure can lead to interference fringes that are challenging to remove through filtering.

### Theoretical simulations

The simulations were performed to mimic the anticipated transformation of TSks into Hopfions by increasing the PMA at the top and bottom of the TSk. Effective medium models work well with simulating thin films because the magnetic properties of the material are relatively uniform over the thickness of the film. To simulate the S30 system, where this assumption of uniformity does not hold, the anisotropy was varied through the film thickness.

As experimentally measured spatial profiles of the PMA of each individual Co layer are not available for our system, we approximated it by an assembly of three magnetic multilayers with the same DMI and the same thickness. The PMA in each of the three subsystems was assigned a spatially varying PMA, e.g., as a trial function, the linearly varying PMA distribution ($K = \frac{3}{{52}} + \frac{6}{{13}}x$), where *x* is the distance from the middle of the central layer, measured in units of the thickness of a layer and PMA being dimensionless. The top and bottom third were assigned the same PMA distribution but with a value that is higher than the PMA in the central third, e.g., 3:1. Given that the actual variation of PMA in S30 is not known, a variety of simulations were performed with multiple profiles of varying the PMA between the central layer and the top/bottom layer.

One set of simulations followed a model published previously^12^ where the central layer has no PMA and the top and bottom layer have a uniform PMA. It was found that above a certain critical value of PMA, the out-of-plane structure of TSks no longer reached the top or bottom layer of the material, but a Hopfion was formed. Below this critical PMA value, only TSks were found to be stable. While this approach helps account for the effects of a reduced PMA due to shape anisotropy, it is likely that the central layer still has a nonnegligible effective PMA, and that the PMA in all layers varies spatially over each layer.

Another set of simulations implemented a linearly varying PMA in all layers, with the PMA value being smallest in the central layer and increasing outward, allowing for a relative PMA ratio between the different PMA layers to be quantitatively described. It was found that when the PMA ratio of the high PMA layer to the low PMA layer is 3:1 or greater, a Hopfion can be stabilized when the out-of-plane structure of the TSk does not extend through the thickness of the material (Fig. 3). Below this ratio, Hopfions are not stabilized. This is consistent with VSM measurements of {Ir(1 nm)/Co(1 nm)/Pt(1 nm)}x10 multilayers compared to {Ir(1 nm)/Co(1.5 nm)/Pt(1 nm)}x10 multilayers showing a PMA ratio of 3–4 between the high (Co=1 nm) and low (Co=1.5 nm) Co film.

None of these simulations included the demagnetizing field (stray field energies), which is an acceptable approximation for several reasons. Compared to TSks, Hopfions generate much weaker stray fields^30^, which means that the addition of stray field energies increases the likelihood of a Hopfion being stabilized energetically. Further, the additional in-plane shape anisotropy generated from the disk structure as a result of the demagnetizing field is accounted for in the simulations by setting the PMA value of the low PMA layers to zero.

The simulated MTXM and X-PEEM patterns shown in Fig. 1c were derived from these simulations using the actual wave vector for the incoming X-rays, i.e., perpendicular to the sample surface for MTXM and therefore only sensitive to the out-of-plane component of magnetization, and at an inclined angle of 30° for X-PEEM, which is sensitive to both in-plane and out-of-plane components of magnetization.

## Supplementary information

Supplementary Information

## Data Availability

The data that support the findings of this study are available from the corresponding author upon reasonable request.
