# Supplementary material for: Creation and observation of Hopfions in magnetic multilayer systems
Source: Nat Commun. 2021 Mar 10;12:1562. doi: 10.1038/s41467-021-21846-5 (PMC7946913; doi:10.1038/s41467-021-21846-5)
Supplement: Supplementary file 1 — Supplementary Information [file 41467_2021_21846_MOESM1_ESM.pdf]

SUPPLEMENTARY INFORMATION

Creation and observation of Hopfions in magnetic multilayer systems

Noah Kent[1,2], Neal Reynolds[1,3], David Raftrey[1,2], Ian T.G. Campbell[1,3], Selven Virasawmy[4], Scott Dhuey[4], Rajesh V. Chopdekar[5], Aurelio Hierro-Rodriguez[6], Andrea Sorrentino[7], Eva Pereiro[7], Salvador Ferrer[7], Frances Hellman[1,3], Paul Sutcliffe[8], Peter Fischer[1,2]

[1]*Materials Sciences Division, Lawrence Berkeley National Laboratory, Berkeley, CA 94720, USA*

[2]*Physics Department, UC Santa Cruz, Santa Cruz CA 95064, USA*

[3]*Department of Physics, University of California, Berkeley, Berkeley, CA 94720, USA*

[4]*The Molecular Foundry, Lawrence Berkeley National Laboratory, Berkeley, CA 94720, USA*

[5]*Advanced Light Source, Lawrence Berkeley National Laboratory, Berkeley, CA 94720, USA*

[6]*Department of Physics, University of Oviedo, 33007 Oviedo, Spain*

[7]*ALBA Synchrotron, 08290 Cerdanyola del Vallès, Spain*

[8]*Department of Mathematical Sciences, Durham University, Durham DH1 3LE, UK*

The supplementary material in this section will provide additional information to support the observation of a magnetic Hopfion in the two multilayer systems studied.

- Experimental X-PEEM data confirm the observation of a TSk spin texture in the sample S7.
- Extended simulations of X-PEEM and MTXM images of possible 3D spin texture in those systems, including torons, Tsk and Hopfions to showcase both similarities and distinct features among those.

S1. X-PEEM images of the S7 multilayer

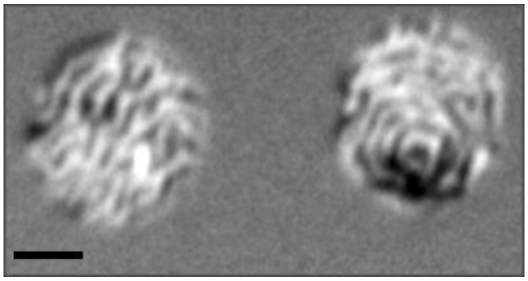

The above image shows the experimentally obvserved domain structure of the S7 multilayer system in a disk with a diameter of 1500nm. It was imaged with X-PEEM at the Co L_3 absorption edge and is found to be similar to the TSk textures observed previously with MTXM[1]. Notably, at the bottom portion of the right disk shown above a 2π TSk is clearly visible. We conclude that S7 is hosting TSks with a characteristic PMA domain structure that extends through the thickness of the nanodisk. The scale bar shown is 500nm.

S2. Extended micromagnetic simulations in magnetic multilayers.

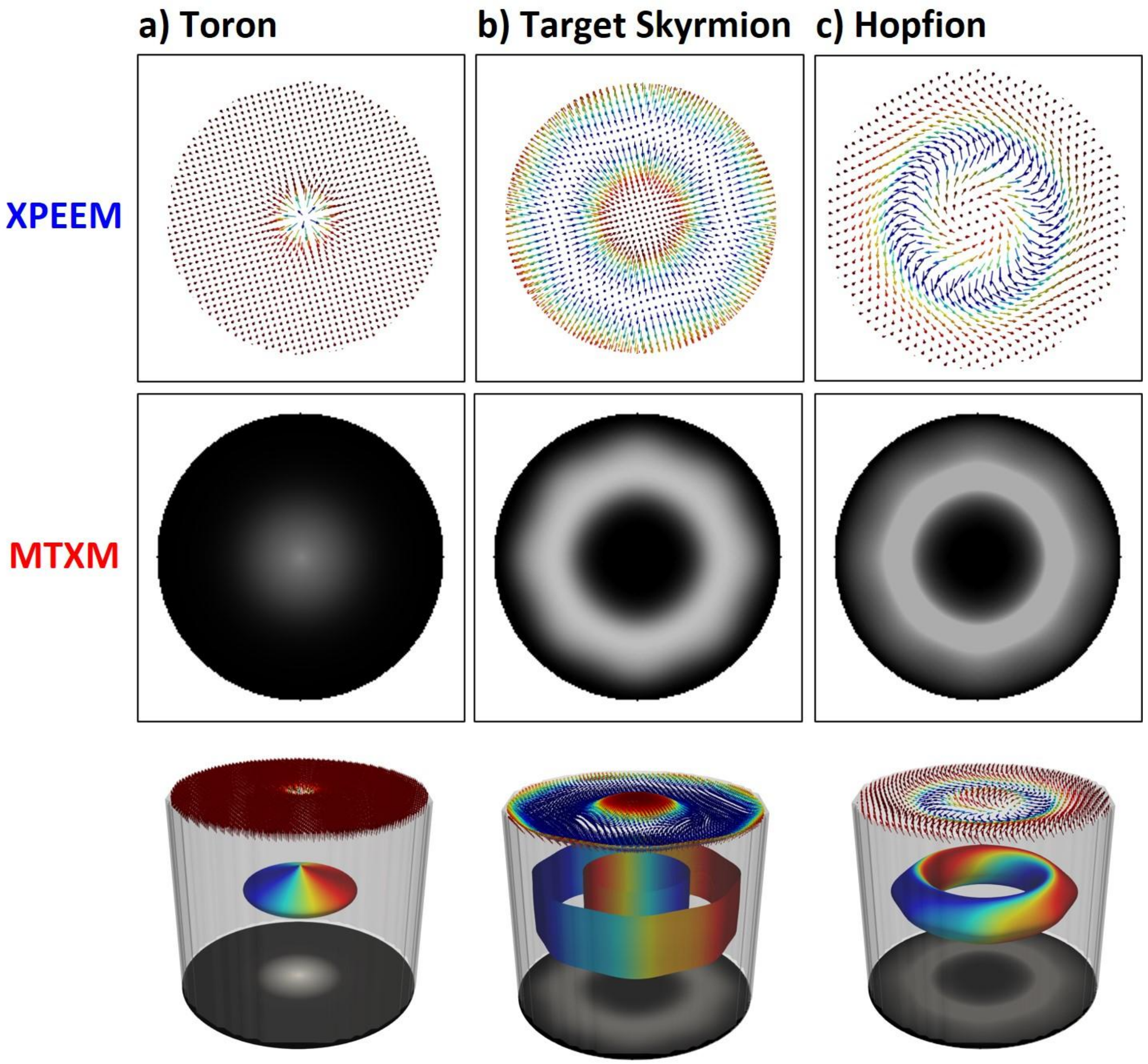

This figure summarizes extended micromagnetic simulations of possible 3D spin textures and their expected appearance in X-PEEM and MTXM images in the magnetic multilayers that were studied in this work. Column a) shows a magnetic toron[2], column b) a target skyrmion, and column c) a Hopfions are displayed. The X-PEEM simulations are shown in the top row and, the MTXM simulations in the central row. The bottom row is an artistic drawing, combining the simulated X-PEEM and MTXM images at the top and the bottom of a cylinder, and the contour of zero out-of-plane magnetization in the center of the cylinder.

a) The magnetic toron shows in the X-PEEM image - apart from a radially symmetric area in the center - a rather uniform magnetization pointing into the opposite direction than in the center. Similarly, the MTXM image is expected to show a relatively small light center surrounded by a uniform magnetization, pointing into the opposite directions (black).

b) The target skyrmion shows the expected characteristic multi-ring structure, which for the MTXM image exhibits a black central area followed by a bright ring and then a wide, black ring at the perimeter. The X-PEEM image shows a similar multi-ring structure as the MTXM, indicative of the multiple rotations of the magnetization into radial outward direction of the disk.

c) The Hopfion structure, although quite similar to the TSk for the MTXM image, exhibits a X-PEEM image that is in stark difference to both the toron and the TSk.

Comparing those three spin textures with the experimental data, c.f. Figs 2c and 2f in the main manuscript, taking into account experimental statistics and noise level in the images, there is no agreement with a toron, but a reasonably good agreement with the Hopfion texture.

References

1 Kent, N. *et al.* Generation and stability of structurally imprinted target skyrmions in magnetic multilayers. *Applied Physics Letters* **115**, 112404 (2019).

2 Leonov, A. O. & Inoue, K. Homogeneous and heterogeneous nucleation of skyrmions in thin layers of cubic helimagnets. *Phys Rev B* **98**, 054404 (2018).

3 Müller, G. P., Rybakov, F. N., Jónsson, H., Blügel, S. & Kiselev, N. S. Coupled quasimonopoles in chiral magnets. *Phys Rev B* **101**, 184405 (2020).
